# The effect of a contact‐based intervention on attitudes and intended behaviors of nursing students toward people with mental illness: A quasi‐experimental study

**DOI:** 10.1002/hsr2.954

**Published:** 2022-11-25

**Authors:** Mohammad A. Q. M. Al Ma'ani, Shaher H. Hamaideh, Ayman M. Hamdan‐Mansour

**Affiliations:** ^1^ Nursing Department, College of Health Sciences University of Fujairah Fujairah United Arab Emirates; ^2^ Community and Mental Health Nursing Department, Faculty of Nursing The Hashemite University Zarqa Jordan; ^3^ Community Health Nursing Department, Psychiatric Nursing, School of Nursing The University of Jordan Amman Jordan

**Keywords:** attitudes, behaviors, contact‐based intervention, mental illness, nursing student

## Abstract

**Background and Aim:**

Although nursing students are professionally and ethically trained and educated to respect patients with a variety of medical and mental problems, they continue to exhibit negative attitudes and behaviors toward mental disease and patients with mental illness. The accumulated evidence indicates that contact‐based intervention (CBI) is helpful in enhancing nursing students' attitudes and behaviors toward patients with mental illness. Although evidence found to support the CBI, culture and settings might play a significant role to decide its effectiveness. This would call for testing further the effectiveness of CBI across cultures and healthcare settings. The purpose of this study was to assess the effect of a CBI on the attitudes and intended behaviors of nursing students in Jordan toward people with mental illness.

**Methods:**

A quasi‐experimental, pre–post, design was used. Data were collected from 81 nursing students from two nursing schools implementing the CBI using self‐reported questionnaires to measure students' attitudes and intended behaviors toward people with mental illness. Data were collected during the first semester of the academic year 2019/2020. The paired‐samples and independent‐samples *t* tests were used to test the study's hypotheses.

**Results:**

At baseline, the results indicated that there were no statistically significant differences between the experimental and control groups in terms of their attitudes and intended behaviors toward people with mental illness. At posttest, statistically significant improvements in the attitudes and intended behaviors of nursing students found in the experimental group and between control and experimental groups toward people with mental illness compared with baseline pretest measures (*p* < 0.001).

**Conclusion:**

The CBI did improve the attitudes and intended behaviors of Jordanian nursing students toward people with mental illness. Significant implications for nurses were discussed.

## 1 INTRODUCTION

Although nursing students are professionally and ethically trained and educated to respect patients with medical and mental problems, they continue to exhibit negative attitudes and behaviors toward mental illness and patients with mental illness.^1^, ^2^ Nursing students have a high level of unfavorable negative attitudes toward patients with mental illness, including an assumption of danger, unpredictability, and a desire for social distance.^3^ These attitudes have found to be detrimental to students' performance including the use of nontherapeutic communication, while caring of patients with mental illness.^4^ These attitudes might contribute to lower quality of nursing care and adversely affecting patient outcomes.^5^, ^6^ This would call for finding out means to combat nursing students' negative attitudes and intended behaviors during their theoretical and clinical training courses.

Three primary strategies for combating negative attitudes and behaviors among students were presented in the literature: protest, education, and contact.^7^ Contact, the most promising strategy, relates to interpersonal interactions between members of the general public and individuals with mental illness,^8^ but protest and education have significant limitations.^7^ Contact‐based intervention (CBI) has also been showed to lessen nursing students' fear, perceived danger, avoidance, segregation, and coercive attitudes toward people with mental illness.^9^ Additional research revealed that CBI resulted in a positive shift in attitudes, an increase in empathy, and a positive shift in the intentions of nursing students to seek a profession in mental health nursing in the future.^10^, ^11^, ^12^, ^13^ The accumulated evidence indicate that CBIs are helpful in enhancing nursing students' attitudes and intended behaviors toward patients with mental illness.^14^ Although evidence found to support the CBI, previous studies are inconsistent and have methodological limitations, as well as lack long‐term follow‐up data on the sustainability of CBI effect.^7^ Furthermore, culture and settings might play a significant role to decide its effectiveness. For example, in Jordan, the healthcare systems, treatment protocols, available facilities for mental health care, and shortage of workforce might influence the recovery process of patients with mental illness, and consequently, nurses and nursing students' perception. This would call for testing further the effectiveness of CBI across cultures and healthcare settings. Furthermore, nursing students found to suffer higher levels of academic anxiety and depressive symptoms due to mandate academic assignments and training needs.^15^, ^16^ Further, studies have also reported that university students, in general, are suffering various forms of psychological disturbances such substance use, deliberate self‐harm, and premenstrual dysphoric disorder^17^, ^18^ that might influence their behaviors and attitudes.

In Jordan, similar to international reports, stigma is observed against mental illness and patients with mental illness and their families.^19^, ^20^ Negative attitudes were also reported among nurses toward patients with mental illness and mental illness,^5^ and among nursing students toward patients with mental illness,^1^ which may indicate the need for intervention to combat negative attitudes and behaviors of nursing students. The challenges that are facing nursing education, nowadays, are also signifying the importance of the study.^21^ Therefore, there is a need to experiment the proposed CBI approach to correct attitudes and intended behaviors among nursing students and nurses. Focusing on nursing students would give an indication for nurse educators and clinicians about the real needs and areas of defects in nursing education and training that need to be corrected. Therefore, this study aims to assess the effect of a CBI on the attitudes and intended behaviors of nursing students in Jordan toward patients with mental illness. The study hypotheses were as follows: (1) In the experimental group, nursing students will report more positive attitudes and intended behaviors toward people with mental illness postintervention compared with the baseline and (2) at the posttest, Jordanian nursing students in the experimental group will report more positive attitudes and intended behaviors toward people with mental illness than control group.

## 2 METHODS

### 2.1 Design

This study utilized a quasi‐experimental nonequivalent control group pretest–posttest design to examine the impact of a CBI on attitudes and intended behaviors of nursing students toward people with mental illness. Data were collected in relation to attitudes and intended behaviors toward people with mental illness using self‐administered questionnaires at two points of time (before and after the CBI).

### 2.2 Sample and settings

Nursing students were recruited from two nursing schools (one public and one private) in Jordan. The two schools selected randomly per cluster (private vs. public). Nursing students recruited conveniently from the two selected schools. Inclusion criteria included: (1) nursing students enrolled regularly, (2) have completed at least one clinical course in nursing. Exclusion criteria included: (1) nursing students who currently taking/have taken mental health nursing courses (theory, clinical, or both) were excluded from this study due to expected effect of training at clinical area on their attitudes toward mental illness and patients with mental illness,^1^, ^22^ and (2) students diagnosed or have a history of mental illness as this might influence their baseline information data and their personal experiences would influence attitudes and intended behaviors.

### 2.3 The sample power

A power analysis was conducted to determine the appropriate sample size using the G*Power computer software program version 3.0.10.^23^ In the current study, a paired‐samples *t* test test was used; a small effect size of 0.25 was determined, a significance level of *α* = 0.05 was set, and at a power of 0.90. The yielded sample was 34 participants, at least, for each group. Subsequently, the overall sample size was 68, at least, for the 2 groups. However, 10% (~7 subjects) of the total sample size were added to account for any refusal and dropout. Accordingly, 81 subjects were recruited (41 in the intervention group and 40 in the control group).

### 2.4 The intervention

In the current study, the implemented CBI included a presentation done by two volunteered persons with mental illness who disclosed mental health challenges, treatment, and recovery to the participated nursing students in the classrooms at the selected nursing schools. The two persons with mental illness, who are mentally stable and hold down jobs, were recruited from a national nonprofit association, which is led by persons with mental illness and is dedicated to support and defend the rights of people with mental illness. Both persons were not socially nor occupationally impaired, their cognitive ability was intact, and their psychological symptoms were remitted. Those people have been invited to meet with nursing students. The persons with mental illness were diverse in term of sex (male and female) and were trained before going to each nursing school where they interacted with the participated nursing students. The implemented CBI included written material in the form of a brochure to disseminate more information in the meeting. In each nursing school, the CBI involves 30 min live interaction session between the two persons with mental illness and nursing students in the classrooms of the nursing schools. In this session, the persons with mental illness provided personal testimonies describing personal perspectives and experiences of having a mental illness. The implemented CBI discussed “on the way down” and “on the way up” stories of the persons with mental illness. “On the way down” stories discussed suffering, illness, and disabilities, whereas “on the way up” stories discussed the way to recovery and success. In addition to the participated nursing students and the persons with mental illness, the implemented CBI involved a psychiatric nurse. The role of the psychiatric nurse was to supervise the implemented CBI by facilitating the interaction between the participated nursing students and the persons with mental illness. In addition, the psychiatric nurse was responsible for conducting the questions–answers session. The psychiatric nurse was recruited to intervene appropriately in the cases of expected emotional distress that might be experienced by the participated nursing students or the persons with mental illness. After that, 15 min for facilitated audience discussion in the form of questions and answers was conducted. Upon completion of the session, the researcher distributed the self‐reported questionnaire to the participating nursing students and answer any related inquiries.

Intervention fidelity is the adherence of an intervention to the protocol. A checklist was used to document the fidelity of the implemented CBI on the members of the intervention group. It measured the pace and timing of the CBI, the engagement of the participated nursing students, and the role of the psychiatric nurse and the persons with mental illness.

### 2.5 Data collection procedure

After obtaining ethical approval from targeted universities and approval from the volunteered charitable institution, data collection started during the first semester of the academic year 2019/2020. The persons with mental illness were recruited from the interested and willing members in the institution. They were informed about the study in details. Consequently, they provided consent to indicate their agreement to take part in this study. Then, they were informed by phone the time and location for each session. The nursing students were recruited through school administrator who volunteer to invite students through contact‐based information system per school. The invitation included contact information of the research team. Interested students were informed about the study purpose and significance. Then, the interested ones directed to meet with the research team to sign the consent form.

In the first week and at each nursing school alone, students were asked to leave the class quietly if they had any mental illness or a history of mental illness. Students who were willing to meet people with mental illness were recruited in the intervention group, whereas students who preferred to receive no intervention but want to take part in the study were directed to the control group. Assigning students to intervention and control group was based on their preference, as some students were willing to interact with the persons with mental illness, while others were not willing. Subsequently, the data were collected by the researcher using the self‐reported questionnaire that included the researcher‐developed questions, Mental Illness: Clinicians' Attitudes (MICA‐4), and Reported and Intended Behaviors Scale (RIBS), to screen the attitudes and intended behaviors of the participated nursing students in both groups toward people with mental illness.

In the second week and at each nursing school alone, the control group was asked to complete the same self‐reported questionnaire filled in the first week. Then, the members of the control group were asked to kindly leave the classrooms before the CBI begins; as a result, only the members of the intervention group stayed in the classrooms. Subsequently, the CBI took place among the members of the intervention group only, under the supervision of the researcher, and lasted around 30 min. After the CBI finished, a 15 min session for questions and answers between the participated nursing students at the intervention group and the persons with mental illness took place, and was facilitated by the researcher. Subsequently, the data were collected by the researcher from the members of the intervention group using the same self‐reported questionnaire that was administered in the first week. The collected data from both groups at 2 weeks were kept in a password‐secured computer.

### 2.6 Ethical considerations

Ethical approvals were obtained from nursing schools and the permission to use the translated Arabic version of MICA‐4^24^ was sought and received. Both the nursing students and the persons with mental illness were informed about the study, the potential benefits and risks, and their rights. Those who agreed to take part in this study provided informed consent.

### 2.7 Instrumentation

Data has been collected using the Arabic version of the tools. Two scales were used in this study, in addition to a researcher‐developed questionnaire to collect the demographic data including age, gender, family month income, university, and previous contact with people with mental illness. The two scales were as follows:

1. The MICA v4 scale^24^ was used to measure the attitudes of the participated nursing students toward people with mental illness. The scale is formed of 16 items and takes about 5 min to be completed. The total score of MICA‐4 is the sum of the scores of its individual items (16 items); 10 of these items were reversed scored. A high total score gives an indication of more negative stigmatizing attitudes toward people with mental illness. The MICA‐4 showed acceptable internal consistency with Cronbach's *α* of 0.72. It further had acceptable convergent validity, good face validity, low rates of missing data, and good readability.^24^ In this study, we have used the translated Arabic version that has been conducted according to the back‐translation method^25^ recommended by the original authors of the MICA‐4.^24^
2. The RIBS:^26^ The RIBS was used to measure the intended behaviors of the participated nursing students toward people with mental illness. It took ~1–2 min to complete and generated a score based on the willingness to have contact with people with mental illness (“In the future, I would be willing to work with someone with a mental health problem”). Scores on the RIBS range from 4 to 20, with higher scores indicating more positive intended behaviors. Within adult groups, the RIBS had a moderate/substantial internal consistency and test–retest reliability. Also, it had strong consensus validity.^26^

### 2.8 Data analysis plan

The Statistical Package for the Social Sciences version 25 software for Windows (IBM Corp.). Subgroup mean substitution method was used to replace the missing data. The missing data were substituted with the mean conditional to the group of participants (intervention and control groups). Selection biases were checked by comparing the groups (intervention and control) baseline demographic characteristics. Further, both groups were compared at the baseline (pretest) in regard to the attitudes and intended behaviors toward people with mental illness. Moreover, the assumptions for the used inferential parametric statistical tests were checked and met. Descriptive statistics (mean and median) and dispersion measure (SD and range) have been used to describe the variables of the study. The paired‐samples *t* test was used to test Hypothesis 1 and the independent‐samples *t* test was used to test Hypothesis 2. Alpha was set at 0.05 level of significance.

## 3 RESULTS

### 3.1 Characteristics of the sample

Out of the 81 nursing students (41 students at the experimental group and 40 at the control group) who consented to participate in the study and completed the pretest assessment, only 73 (90%) nursing students completed the posttest assessment. Eight (10%) nursing students did not complete the posttest assessment due to personal reasons; 3 (4%) were in the experimental group and 5 (6%) were in the control group. For the 2 groups, 52 students (64.2%) were female. The mean age was 19.51 years (SD = 0.88), ranging from 18 to 22 years. Sixty‐nine students (85.2%) were sophomores (second year) and 12 (14.8%) were juniors (third year). See Table 1.

**Table 1:** The demographic characteristics of the sample (*N* = 81)

Characteristic	Category	*n*	%
Group membership	Experimental	41	0.51
	Control	40	0.49
Gender	Male	29	35.8
	Female	52	64.2
Academic level	Sophomores	69	85.2
	Juniors	12	14.8

In the experimental group, 29 students (70.7%) were females. The mean age was 19.15 years (SD = 0.76), ranging from 18 to 22 years. All the students were sophomores. In the control group, 23 students (57.5%) were females. The mean age was 19.90 years (SD = 0.84), ranging from 18 to 22 years. Twenty‐eight students (70%) were sophomores.

### 3.2 Comparing attitudes and intended behaviors between both groups at the baseline

An independent‐samples *t* test was performed to find out if Jordanian nursing students in the experimental group statistically and significantly differ in their attitudes and intended behaviors toward people with mental illness from those in the control group, at the baseline measure (pretest). The results revealed that there were no statistically significant differences between experimental (for attitudes: *M* = 48.21, SD = 6.95; for intended behaviors: *M* = 13.58, SD = 4.13) and control groups (for attitudes: *M* = 48.62, SD = 7.85; for intended behaviors: *M* = 14.15, SD = 3.88); for attitudes: *t*(79) = −0.24, *p* = 0.80, two‐tailed; for intended behaviors: *t*(79) = −0.63, *p* = 0.52, two‐tailed (Table 2).

**Table 2:** The differences between the experimental and control groups regarding the attitudes and intended behaviors toward people with mental illness, at the baseline measure (pretest)

Group	Attitudes				Intended behaviors			
	M	SD	*t*	*p*	M	SD	*t*	*p*
Experimental	48.21	6.95	−0.24	0.80	13.58	4.13	−0.63	0.52
Control	48.62	7.85			14.15	3.88		

### 3.3 Effect of CBI on nursing students in the experimental group

To test Hypothesis 1, a paired‐samples *t* test was performed to find out if nursing students, in the control group, are different in their attitudes and intended behaviors toward people with mental illness at pretest and the posttest. All the assumptions of the paired‐samples *t* test were checked and met before conducting the analysis. The results (see Table 3) revealed that there were no statistically significant differences in the attitudes (*t* = 0.02, *p* = 0.97) and intended behaviors toward people with mental illness (*t* = −1.23, *p* = 0.22) of nursing students in the control group. The analysis showed that mean score of attitudes, at baseline and posttest toward people with mental illness, were 48.9 (SD = 8.1) and 48.8 (SD = 9.4), respectively. Regarding intended behaviors, mean at baseline and posttest were *M* = 14.1 (SD = 4.1) and 15.0 (SD = 3.3), respectively.

**Table 3:** The comparison of the attitudes and Intended behaviors of the members of the control and experimental groups between the pretest and posttest

Group	Dependent variable	Pretest		Posttest		*t*	*p*
		M	SD	M	SD		
Control	Attitudes	48.88	8.07	48.82	9.44	0.02	0.97
	Intended behaviors	14.11	4.12	15.02	3.34	−1.23	0.22
Experimental	Attitudes	48.55	6.97	42.73	7.32	4.04	<0.001
	Intended behaviors	13.57	4.26	16.65	3.04	−3.96	<0.001

Further, a paired‐samples *t* test was performed to find out if nursing students, who were in the experimental group, statistically and significantly differ in their attitudes and intended behaviors toward people with mental illness, between the baseline measure (pretest) and the posttest. All the assumptions of the paired‐samples *t* test were checked and met before conducting the analysis. The results revealed that there were statistically significant improvements in the attitudes (*t* = 4.04, *p* < 0.001) and intended behaviors (*t* = −3.96, *p* < 0.001) toward people with mental illness in the experimental group. The analysis showed that mean scores of attitudes, at baseline and posttest toward people with mental illness, were 48.6 (SD = 7.0) and 42.7 (SD = 4.3), respectively. Regarding intended behaviors, mean scores at baseline and posttest were *M* = 13.6 (SD = 4.3) and 16.7 (SD = 3.0), respectively.

### 3.4 Testing differences between experimental and control group

To test Hypothesis 2, an independent‐samples *t* test was performed to find out if nursing students in experimental and control group are different at the posttest in their attitudes and intended behaviors toward people with mental illness. All the assumptions of the paired‐samples *t* test were checked and met before conducting the analysis. The results (see Table 4) revealed that there were statistically significant differences, at the posttest, between experimental (for attitudes: *M* = 42.73, SD = 7.32; for intended behaviors: *M* = 16.65, SD = 3.04) and control groups (for attitudes: *M* = 48.82, SD = 9.44; for intended behaviors: *M* = 15.02, SD = 3.34); for the attitudes, *t* = −3.09, *p* = 0.003, two‐tailed; and intended behaviors toward people with mental illness, *t* = 2.17, *p* = 0.03).

**Table 4:** Differences between the experimental and control groups regarding the attitudes and intended behaviors toward people with mental illness, at the posttest

Group	Attitudes				Intended behaviors			
	M	SD	*t*	*p*	M	SD	*t*	*p*
Experimental	42.73	7.32	−3.09	0.003	16.65	3.04	2.17	0.03
Control	48.82	9.44			15.02	3.34		

## 4 DISCUSSION

Negative attitudes of nursing students toward patient with mental illness and mental illness, in general, has negative impact on quality of care provided and do negatively influence nursing students' preference to specialize in psychiatric care. This study proposed an intervention that combat nursing students' negative attitudes and intended behaviors. We found that using CBI has improved (minimized) nursing students' negative attitudes and intended behaviors toward patient with mental illness and mental illness. The improvement (being less negative) in attitudes and intended behaviors have been observed among students in experimental groups at pre‐ and postintervention and between control and experimental groups at both times (pre and post). This indicates that CBI is promising approach that can be applied at nursing schools as one preparatory stage for students before sending them to psychiatric care settings. The results support previous reports that CBI had positive influence on nursing students' attitudes and behaviors.^9^, ^12^ The study emphasized the importance of introducing such an experience for nursing students for number of benefits including students' preference to intention to pursue a career in mental health nursing in the future that has been also reported by Happell et al.^10^ Also, fear and stereotypical and negative assumptions toward patients with mental illness and mental illness have been counteracted.^9^ Although some researchers reported that nursing students' negative attitudes and behaviors toward patients with mental illness and mental illness have been improved after the course of mental health (theory and practice),^22^ such experience might have number of negative consequences and considered risky to students and the patients. Having the students live the experience will not ensure what has contributed and how students did and what have they used to improve their attitudes and behaviors, and the consequences on the quality of training and care are not assured. The quality of nursing education is mandating maintaining patient safety during training courses and students have the right to receive appropriate preparation before being sent to training area. This is why high‐fidelity simulation has been proposed in nursing education and found to be successful and did enhance patients' safety and quality of nursing education and training.^27^

The option whether to use simulation lab or using the CBI as approaches is of equivalent benefits to students and patients. This is also similar to what has been reported by researcher who have used both types of social contact (Live and video‐based) between nursing students and people with mental illness and had positive outcomes.^28^ Itzhaki et al.^11^ have also found that a mental health nursing course that involved face‐to‐face meetings with three individuals with mental illness and a film on a doctoral degree holder who talked about living with mental illness, resulted in improvement of the attitudes of nursing students toward patients with mental illness. Other researcher^29^ found that nursing students who attended either live or DVD‐based contact reported more positive attitudes and behaviors toward people with mental illness than nursing students who attended a lecture about stigma of mental illness. No significant differences were found between the live and DVD's groups. The inconsistent findings of these studies might be attributed to the fact that these studies have used different measures to assess the attitudes and behaviors of nursing students toward people with mental illness. These studies also have not used identical components of the live and video‐based social contact interventions; some variations have existed. Therefore, it seems that it is not possible to determine which type of social contact is more effective in improving the attitudes and behaviors of nursing students toward people with mental illness.

One limitation of this study is related to using pre–post only. A long‐term follow‐up assessment would be more informative. Previous research reported dearth of long‐term follow‐up data and insights on how to sustain positive changes for longer period of time.^7^ One another limitation is the unavailability of a measure for actual behaviors toward people with mental illness. Also, recruiting the persons with mental illness was challenging. Excluding the students who has/had mental illness would also limit the generalizability of the study findings to this category.

### 4.1 Implications for nursing practice

The current results have significant practice implications for nursing. One of the potential practical implications is that students will provide compassionate mental health care during their practicum and later as independent nurses.^30^ Another possible practice implication is for people with mental illness. The positive changes in attitudes and intended behaviors found in this study may encourage those people to seek additional healthcare and receive appropriate treatment for their physical complaints.^31^

Fighting the stigmatizing attitudes and behaviors is not the battle of nurse researchers only; all practicing nurses, regardless of the level and area of practice, should also be recruited. Nurses in practice should be actively involved in antistigma projects and campaigns that develop and promote CBIs and other stigma‐reduction measures.^32^ Practicing nurses in university‐affiliated hospitals and clinics should screen the attitudes and behaviors of nursing students toward people with mental illness on their practice routine and correct negative stigmatizing ones. Further, nurses who work in university‐affiliated health facilities should develop tailored CBIs and other antistigma interventions for university students. Nurses with mental illness who are willing and comfortable with sharing their own experiences of having a mental illness, may participate in CBIs and antistigma educational interventions simultaneously. This might magnify the effect of stigma reduction,^32^ given that people trust nurses and view nursing as one of the most ethical and honest professions.^33^

Finally, no additional practice suggestions can be provided until more rigorous data becomes available.^32^ However, continued attempts of nurses to address the negative stigmatizing attitudes and behaviors toward people with mental illness, through CBIs and other antistigma approaches, would eventually lead to correcting these negative attitudes and behaviors.

## 5 CONCLUSION

The study found that CBI is a promising approach that can be adopted at nursing education. The positive impact of CBI on nursing students' attitudes and intended behaviors has been sustained. Such approach and similar other approaches have to be addressed and sustained by mental health nursing educators. Faculty members and mental health nursing educators and tutors should be aware about the need of integrating CBI and relevant approaches into nursing curricula. The serious negative impact of nursing students and nurses' attitudes and behaviors toward mental illness and patients with mental illness is a strong indication to improve and modify the nursing education and training to meet the increasing demand of safe and quality psychiatric care practices. More study is needed to compare and test the long‐term effect of CBI and equivalent approaches for feasibility and utility in nursing education settings.

## AUTHOR CONTRIBUTIONS

**Mohammad A. Q. M. Al Ma'ani**: Conceptualization; formal analysis; investigation; methodology; visualization; writing – original draft. **Shaher H. Hamaideh**: Methodology; writing – review & editing. **Ayman M. Hamdan‐Mansour**: Project administration; supervision. All authors have read and approved the final version of the manuscript.

## CONFLICT OF INTEREST

The authors declare no conflict of interest.

## Data Availability

The data that support the findings of this study are available from the corresponding author upon reasonable request.
